# The Impact of Paternal and Maternal Smoking on Semen Quality of Adolescent Men

**DOI:** 10.1371/journal.pone.0066766

**Published:** 2013-06-26

**Authors:** Jonatan Axelsson, Lars Rylander, Anna Rignell-Hydbom, Karl Ågren Silfver, Amelie Stenqvist, Aleksander Giwercman

**Affiliations:** 1 Reproductive Medicine Centre, Skåne University Hospital, Malmö, Sweden; 2 Division of Occupational and Environmental Medicine, Lund University, Lund, Sweden; University Hospital of Münster, Germany

## Abstract

**Background:**

Maternal smoking during pregnancy has been reported to negatively impact sperm counts of the sons. Sufficient data on the effect of paternal smoking is lacking.

**Objectives:**

We wished to elucidate the impact of maternal and paternal smoking during pregnancy and current own smoking on reproductive function of the male offspring.

**Methods:**

Semen parameters including sperm DNA integrity were analyzed in 295 adolescents from the general population close to Malmö, Sweden, recruited for the study during 2008–2010. Information on maternal smoking was obtained from the Swedish Medical Birth Register, and regarding own and paternal smoking from questionnaires. The impacts of maternal, paternal and own smoking were evaluated in a multivariate regression model and by use of models including interaction terms. Totally, three exposures and five outcomes were evaluated.

**Results:**

In maternally unexposed men, paternal smoking was associated with 46% lower total sperm count (95%CI: 21%, 64%) in maternally unexposed men. Both paternal and maternal smoking were associated with a lower sperm concentration (mean differences: 35%; 95%CI: 8.1%, 55% and 36%; 95%CI: 3.9%, 57%, respectively) if the other parent was a non-smoker. No statistically significant impact of own smoking on semen parameters was seen.

**Conclusions:**

Prenatal both maternal and paternal smoking were separately associated with some decrease in sperm count in men of whom the other parent was not reported to smoke.

## Introduction

In a number of published studies, maternal smoking during pregnancy has been associated with lower sperm numbers in exposed sons [1]–[6], although no such association was found in some reports [7]–[9]. All of these were recently reviewed and a good evidence for an association was concluded [10]. Discrepancies between the studies may relate to the source of information regarding smoking habits, the number of cigarettes per day and whether the smoking took place early in pregnancy, a crucial period for the developing male foetal gonad [11].

In Sweden, information on maternal smoking in gestational week 8 to 12 can be derived from the Medical Birth Register (MBR) [12] which is missing in only 3–7% of cases and has a low error rate [13]. Most of the studies regarding impact of maternal smoking during pregnancy on semen quality of the offspring are based on questionnaires given to the sons. The degree of “recall bias” when the information is collected with a 20 years delay is unknown.

No apparent effect of paternal smoking on reproductive function of the sons has yet been reported [2]–[5]. However, smoking has damaging effect on DNA [14]–[16], and the DNA of spermatozoa has been reported to be much more sensitive to damage than DNA of oocytes [17], [18]. Paternal smoking has been reported to cause DNA adducts in embryos [19] of the same type found in sperm of smoking men [20], DNA breaks in cord blood of the offspring [21], and also seems associated with; lower pregnancy rates at assisted reproduction [22], pregnancy loss [23], [24], malformations [25]–[30] and cancer [31]–[36] as well as with reduced birth weight [37] in the offspring. Further, DNA mutations in mice, caused by general air pollution is reported to be inherited with predominance through the paternal germ line [38].

We wanted to validate the information regarding maternal smoking habits during pregnancy by comparing the questionnaire data with those obtained from the MBR, as well as to elucidate the effects of different types of exposure to smoking on sperm parameters, including DNA integrity. Thus, we collected semen samples from 295 men from the general Swedish population and obtained both MBR data on maternal smoking in early pregnancy and additional questionnaire-based information concerning both maternal and paternal smoking during pregnancy and current own smoking.

## Materials and Methods

### Recruitment

In 2008–2010 in Sweden, around 25% of all 18 year-old Swedish men underwent a medical health examination which is part of the enrolment in the military service. All 1681 men who underwent the examination from 1st December 2008 to 27th May 2010, who lived within 60 km from the city of Malmö and were born and raised in Sweden with mothers born and raised in Sweden, were asked to participate. Out of these, 241 accepted and joined the study, giving a participation rate of 14%. To reach a planned number of about 300 participants as in a similar study [39] performed in 2000–2001, an additional sub-cohort of 73 men, 17–20 years old and who fulfilled the other criteria mentioned above, was recruited through advertisement in schools and as friends of participants. Out of totally 314 recruited men, born 1989–1992, two men who smoked, but only cannabis or pipe and only intermittently were excluded due to classification difficulties concerning own smoking. Another two men were excluded due to missing sperm concentrations. Both of these men had extremely low semen volumes of 0.1 mL and 0.2 mL, and both reported spillage during sample collection. Another 15 men had missing abstinence times and were also excluded, giving 295 remaining men. All subjects were paid 500 SEK (55 Euro) for their participation and signed an informed consent.

Twenty-five men were 17 years old, 245 men 18 years old, 21 men 19 years and one man 20.

Mean age was 18 years (SD: 0.41 years) BMI was 23 kg/m^2^ (SD: 3.1 kg/m^2^).

### Ethics statement

The study was approved by the ethical committee of Lund University.

### Semen analysis

The men were asked to keep 48–72 h of abstinence, which 38% did, but were included also if the recorded length was outside of the range. They delivered a semen sample in a room at the laboratory. The samples were analysed according to the WHO guidelines from 1999 [40] and the manual on Basic Semen Analysis of the European Society of Human Reproduction and Embryology [41]. Sperm concentration was assessed by use of an improved Neubauer haemocytometer. Positive displacement pipettes were used for proper dilution of the ejaculate. The laboratory used serves as a reference unit for the external quality control of the European Society of Human Reproduction and Embryology, and the Nordic Association for Andrology.

The SCSA technique has been described in details elsewhere [42]. Briefly, DNA breaks are measured as the susceptibility of sperm DNA to denaturation by an acid. After addition of acridine orange that binds to single-stranded DNA in cells with DNA fragmentation, a red fluorescence is emitted but when acridine binds to non-fragmented and thus double-stranded DNA, a green fluorescence is emitted. The DFI constitutes the proportion of red sperms which are those with an impaired DNA integrity.

### Genital examination

The testicles of the men were palpated concerning consistence and position, and varicoceles were registered and graded (File S1). Twenty-five men had a varicocele at the examination but no man had previously got this diagnosis. Six men had been surgically treated for cryptorchidism and eight men were born with one or both testicles undescended but had experienced a spontaneous descent. No man had cryptorchidism at the clinical examination.

### Questionnaire

All participants filled in a questionnaire about maternal and paternal smoking during pregnancy, current own smoking and indoor exposure to parental smoking during childhood (Table 1) as well as about age, BMI and personal history of Chlamydia trachomatis, gonorrhoea, epididymitis and mumps orchitis, surgically operated varicocele and scrotal trauma (File S1), since these factors could be potential confounders. The questionnaire, which also included questions on a history of cryptorchidism and having made a partner pregnant, has been used in previous studies [39], [43] although questions on paternal smoking were novel.

**Table 1:** Prevalence (%) of tobacco exposure (current own smoking, maternal and paternal smoking during pregnancy and indoor tobacco smoke exposure as a child) in 295 17–20 year-old men from the general population, Malmö, Sweden, 2008–2010.

	Current own smoking	Maternal smoking		Paternal smoking	Indoor childhood
	Questionnaire data	Questionnaire data	MBR data	Questionnaire data	Questionnaire data
	n = 295	n = 287^b^	n = 283^c^	n = 278^d^	n = 291^e^
No	233	234	213	198	225
Yes	62	53	70	80	66
1–9 cigarettes/day	37^a^	NA	36	NA	NA
>9 cigarettes/day	20^a^	NA	34	NA	NA

Abbreviations: MBR, medical birth register; NA, information not available.

a Number of cigarettes were below one per day in three men, missing in three men and reported as one per day in one man that reported himself as a non-smoker;

b Missing in eight men;

c Missing in twelve men;

d Missing in 17 men;

e Missing in four men.

### Register-based data on maternal smoking

Data from the MBR on maternal smoking in gestational week 8–12 is recorded by a midwife that questions the woman at first visit in maternity ward. This data on the included men was achieved from the MBR at The National Board of Health and Welfare, through the personal identification numbers of the participating sons (File S1). In this register no information about paternal smoking is recorded.

### Statistical methods

Primarily, the consistency between information on maternal smoking derived from questionnaires and MBR was assessed using the Kappa statistic.

We thereafter used MBR as the source of information on maternal smoking, except for the DFI analysis which was performed after linking of our data with the MBR. Since the merged dataset was coded, we could not relate SCSA results to the MBR based information on maternal smoking.

The data on paternal and own smoking were available from questionnaires only.

For sperm concentration, total sperm count, semen volume, progressive sperm motility and DFI, crude means and standard deviations as well as medians and ranges were first calculated. This was also done for each category of parental smoking during pregnancy with crude values for unexposed and exposed.

Associations between smoking exposures and semen parameters were studied by use of linear regression models. Due to skewed distributions of the residuals, sperm concentration and total sperm count were transformed by use of the natural logarithm. In all analyses concerning semen parameters, abstinence time, divided in five categories: <48, 49–72, 73–96, 97–120 and >120 hours, was considered as a potential confounding factor. Since the number of men reporting an infectious genital disease was low, with only three men having a history of Chlamydia trachomatis, three with epididymitis and none with gonorrhoea or mumps orchitis, we did not adjust for these variables in the analyses.

Primarily we performed univariate analyses testing all the main tobacco exposure modes (own smoking; maternal smoking, paternal smoking) one by one, adjusting for abstinence time as above.

Secondly, a multivariate linear regression analysis was performed with inclusion of all three exposure modes at the same time and abstinence time as above.

In addition, we included in the model, one at a time, the following interaction terms: “paternal smoking*maternal smoking”, “paternal smoking*own smoking”, and “maternal smoking*own smoking”. If statistically significant interaction was found, 2×2 tables were created and vertical as well as horizontal statistical significances were tested in a linear regression model adjusted for abstinence time and the third type of smoking exposure.

In order to test the robustness of our results regarding the impact of paternal smoking following modifications of the non-interaction multivariate analysis were done:

- maternal smoking as reported in the questionnaire instead of MBR;
- adjusting for abstinence time as a continuous variable;
- adjusting for the extent of own or maternal smoking (none, 1–9 or > = 10 cigarettes per day);
- including potential confounders such as indoor parental smoking during childhood, age, BMI, varicocele at examination, previous testicular trauma with discoloration and swelling,
- use of cubic root transformed total sperm count and sperm concentration instead of ln-transformed values, as suggested by some authors [44].

We did not adjust for a history of cryptorchidism, since it might be a part of the pathogenetic pathway linking paternal smoking to changes in semen quality [45], [46], but our study was not designed to ensure top quality data regarding undescended testes.

IBM SPSS Statistics version 18 was used for statistic calculations.

## Results

### Questionnaire vs. MBR data on maternal smoking

Eighty-three percent of the mothers who smoked according to the questionnaire were registered as smokers in MBR, whereas 61% of the mothers registered as smokers in MBR were reported as such in the questionnaire. The Kappa value between the questionnaire and MBR data was 0.63 (p<0.001), (95% CI: 0.51, 0.74), based on 276 cases. Eight men were unable to answer the question about their mother's smoking habits and for 12 men data in MBR were missing.

### Exposure to smoking and semen parameters

The prevalence of smokers and extent of smoking in the different exposure modes are shown in Table 1. Numbers of men with more than one mode of exposure depending on own smoking are shown in table 2. Crude means, standard deviations, medians and ranges for semen variables in all men and depending on exposure during pregnancy are shown in table 3.

**Table 2:** Number of men with different exposures to parental smoking during pregnancy in 295 17–20 year-old men from the general population, Malmö, Sweden, 2008–2010.

	Maternal and paternal (n)	Maternal only (n)	Paternal only (n)	Neither maternal nor paternal (n)
**Participant current smoker** a (n = 55)	8	6	9	32
**Participant not current smoker** a (n = 211)	26	24	31	130

a Information about parental smoking was missing in 7 smoking men and 22 non-smoking men.

**Table 3:** Semen variables and abstinence time as crude values, before any transformation and adjustment, in all men and in relation to parental smoking during pregnancy in 295 young men from the general population, Malmö, Sweden, 2008–2010.

	All men (n = 295)		Mother No (n = 213)^a^		Mother Yes (n = 70)^a^		Father No (n = 198)		Father Yes (n = 80)	
	Mean [SD]	Median [range]	Mean [SD]	Median [range]	Mean [SD]	Median [range]	Mean [SD]	Median [range]	Mean [SD]	Median [range]
**Sperm concentration** (×10^6^/mL)	71 [60]	56 [0.0–410]	76 [65]	61 [0.0–410]	57 [44]	52 [0.08–200]	76 [64]	60 [0.0–410]	61 [50]	53 [0.6–220]
**Semen volume** (mL)	2.9 [1.5]	2.7 [0.40–14]	2.9 [1.5]	2.8 [0.40–9.1]	3.0 [1.8]	2.7 [0.70–14]	3.1 [1.6]	2.8 [0.40–14]	2.6 [1.4]	2.3 [0.70–9.1]
**Total sperm count** (×10^6^)	206 [215]	150 [0.0–1700]	230 [240]	160 [0.0–1700]	160 [130]	130 [0–590]	230 [240]	170 [0.0–1700]	150 [130]	120 [2.0 to 590]
**Progressively motile sperm** (%)^b^	53 [17]	58 [0.0–86]	53 [17]	58 [0.00–86]	52 [15]	56 [6.0–77]	54 [15]	58 [0.00–86]	49 [17]	52 [2.0–77]
**DFI** (%)^c^	11 [6.2]	10 [3.0–39]	11 [6.5]	10 [3.0–39]	10 [4.8]	9.5 [3.0–25]	11 [6.4]	10 [3.00–39]	11 [5.4]	10 [3.0–29]
**Abstinence time** (h)	60 [34]	58 [8.0–230]	63 [33]	59 [10–230]	52 [37]	47 [8.0–230]	64 [35]	59 [10–230]	52 [30]	52 [8.0–140]

Abbreviations: DFI, DNA fragmentation index; SD, standard deviation.

a For DFI: Mother No n = 229, Mother Yes n = 52.

b Missing in one man.

c Missing in six men.

The results of the primarily performed, univariate analyses, adjusted only for abstinence time, are shown in Table 4 and 5, and showed a 33% lower total sperm count (95%CI: −50%, −9.5%) and 4.8 percentage points lower fraction of progressively motile sperm (95%CI: −9.2, −0.35) in men reporting exposure to paternal smoking at the time of pregnancy. Furthermore, sperm concentration had a tendency to be lower in paternally exposed men (−23%, 95%CI: −42%, 1.0%). Own smoking and maternal smoking were not significantly associated with semen parameters, but sperm concentration and total sperm count tended to be lower in maternally exposed men (−23%, 95%CI: −43%, 3.3% and −22%, 95% CI: −43%, 8.0%, respectively) (table 5).

**Table 4:** Mean differences and 95% confidence intervals of difference [95%CI] for untransformed semen variables according to type of exposure (yes compared to no) and adjustment in 295 17–20 year-old men from the general population, Malmö, Sweden, 2008–2010.

	Own smoking						Maternal smoking						Paternal smoking					
Adjustment:	Univariate^a^			Multivariate^b^			Univariate^a^			Multivariate^b^			Univariate^a^			Multivariate^b^		
	n = 295			n = 277			n = 283			n = 277			n = 278			n = 277		
	MD	95%CI	p	MD	95%CI	p	MD	95%CI	p	MD	95%CI	p	MD	95%CI	p	MD	95%CI	p
**Semen volume** (mL)	−0.39	−0.81, 0.035	0.072	−0.34	−0.80, 0.12	0.15	0.17	−0.24, 0.59	0.41	0.35	−0.10, 0.81	0.13	−0.35	−0.75, 0.049	0.085	−0.51	−0.94, −0.070	0.023
**Progressive motility** (%)^c^	3.2	−1.6, 8.0	0.20	2.8	−2.3, 7.8	0.28	−0.076	−4.8, 4.6	0.98	0.47	−4.5, 5.5	0.85	−4.8	−9.2, −0.35	0.034	−4.3	−9.1, 0.48	0.077
**DFI** (%)^d^	−0.91	−2.6, 0.79	0.29	−0.63	−2.5, 1.3	0.52	−0.79	−2.7, 1.1	0.41	−0.73	−2.8, 1.3	0.48	−0.35	−2.0, 1.3	0.67	−0.009	−1.8, 1.8	0.99

Abbreviations: 95%CI, 95% confidence interval; DFI, DNA fragmentation index; MD, mean difference.

a Adjusted for abstinence time.

b Adjusted for abstinence time, current own smoking and maternal and paternal smoking during pregnancy.

c Missing in one man.

d Missing in six men.

**Table 5:** Mean relative (%) differences and 95% confidence intervals of difference [95%CI] in back-transformed semen variables, according to type of exposure (yes compared to no) and adjustment in 295, 17–20 year-old men from the general population, Malmö, Sweden.

	Own smoking						Maternal smoking					Paternal smoking						
Adjustment:	Univariate^a^			Multivariate^b^			Univariate^a^			Multivariate^b^			Univariate^a^			Multivariate^b^		
	n = 293			n = 264			n = 292			n = 264			n = 276			n = 264		
	MD	95%CI	p	MD	95%CI	p	MD	95%CI	p	MD	95%CI	P	MD	95%CI	p	MD	95%CI	P
**Sperm concentration**	2.9%	−25%, 41%	0.86	0.10%	−27%, 37%	0.10	−23%	−43%, 3.3%	0.080	−12%	−35%, 20%	0.41	−23%	−42%, 1.0%	0.059	−18%	−39%, 11%	0.20
**Total sperm count**	−4.0%	−31%, 34%	0.81	−4.4%	−32%, 34%	0.80	−22%	−43%, 8.0%	0.14	−4.9%	−32%, 33%	0.77	−33%	−50%, −9.5%	0.009	−31%	−50%, −4.9%	0.024

Abbreviations: 95%CI, 95% confidence interval; MD, mean difference.

a Adjusted for abstinence time.

b Adjusted for abstinence time, current own smoking and maternal and paternal smoking during pregnancy.

When, the multivariate analysis was performed, including current own smoking, maternal and paternal smoking during pregnancy, and abstinence time in the models (Table 4 and 5), total sperm count was 31% lower (95%CI: −50%, −4.9%) and semen volume was 0.51 mL lower (95%CI: −0.94, −0.070) in men exposed to paternal smoking during pregnancy than in men that were not. This corresponded to a total sperm count of 120×10^6^ if exposed versus 180×10^6^ if unexposed.

No other statistically significant differences were found.

We found statistically significant interactions between paternal smoking and maternal smoking, for both total sperm count (p = 0.034) and sperm concentration (p = 0.024). Thus, both sperm concentration (mean difference: −35%; 95%CI: −55%, −8.1%; p = 0.016) and total sperm count (mean difference: −46%; 95%CI: −64%, −21%; p = 0.002) were decreased in paternally exposed men, but only if their mothers were non-smokers (Table 6 and 8). For men with smoking mothers, paternal smoking did not have any significant impact on the sperm numbers. Maternal smoking was associated with a lower sperm concentration only in paternally unexposed men (mean difference: −36%; 95%CI: −57%, −3.9%, p = 0.031) (Table 7 and 9).

**Table 6:** Impact of paternal smoking on sperm concentration depending on maternal smoking status (+/−).

	Paternal smoking	
Maternal smoking	+	−
**+**	+39% (−21%, 140%), p = 0.25	Reference
**−**	−35% (−55%,−8.1%), p = 0.016	Reference

The numbers indicate mean relative (%) differences (95% confidence interval of mean differences) and p-values.

**Table 7:** Impact of maternal smoking on sperm concentration depending on paternal smoking status (+/−).

	Paternal smoking	
Maternal smoking	+	−
**+**	+34% (−18%, 120%), p = 0.24	−36% (−57%, −3.9%), p = 0.031
**−**	Reference	Reference

The numbers indicate mean relative (%) differences (95% confidence interval of mean differences) and p-values.

**Table 8:** Impact of paternal smoking on total sperm count depending on maternal smoking status (+/−).

	Paternal smoking	
Maternal smoking	+	−
**+**	15% (−36%, 110%), p = 0.64	Reference
**−**	−46% (−64%, −21%), p = 0.02	Reference

The numbers indicate mean relative (%) differences (95% confidence interval of mean differences) and p-values.

**Table 9:** Impact of maternal smoking on total sperm count depending on paternal smoking status (+/−).

	Paternal smoking	
Maternal smoking	+	−
**+**	45% (−13%, 140%), p = 0.15	−31% (−56%, 7.7%), p = 0.10
**−**	Reference	Reference

The numbers indicate mean relative (%) differences (95% confidence interval of mean differences) and p-values.

The statistically significant associations found for paternal smoking in the regular multivariate analysis were robust to additional adjustments as mentioned under “Statistical methods” except for the impact of paternal smoking on semen volume, for which no longer any statistically significant difference was seen when we adjusted for the questionnaire data on maternal smoking. (data not shown). Maternal and own smoking in the regular multivariate analysis remained without any statistically significant effect on semen parameters after the adjustments (data not shown).

## Discussion

The most significant finding of this study of adolescent men from the general population, was a reduced total sperm count in men whose father smoked at the time of the pregnancy. This was robust to adjustment for own smoking and for exposure during early childhood, but was only present in men with non-smoking mothers. We also found a lower sperm concentration in men if either the mother or the father smoked and the other parent did not. An additional finding was an underreporting of maternal smoking in questionnaires as compared to MBR data. Still, there was an agreement between the two, with a kappa value of 0.63, which can be interpreted as moderate to substantial [47], [48].

We believe that this cohort is fairly representative of the general Swedish adolescent male population. Since participation rate was only 14%, one may argue for a selection bias related to reproductive capacity but this seems somewhat unlikely, since pregnancy rates are as low as 3% in Swedish 15–19 year-old women [49] and only 19 (6.4%) out of the 295 included men reported to have made a partner pregnant and 11 men (3.7%) to have had regular unprotected intercourse during at least a year without causing a pregnancy. Further, similar semen parameters have been found in conscripts and men recruited through schools or through other participants [50]. In addition to this, a previous study in military conscripts showed that men that provided semen samples for a study with a similarly low participation rate did not differ as considers levels of inhibin B and follicle-stimulating hormone from men who denied to deliver an ejaculate [43]. Since these hormones are markers of spermatogenesis [51], the men that provided semen samples were considered representative of the total group of conscripts [43].

Previous studies regarding impact of paternal smoking on semen quality of the offspring are few [2]–[5] and, in contradiction to our results, did not show any statistically significant association. Only two, however, studied the association with total sperm count [2], [5]. One of the explanations to not finding an association might be an insufficient statistical power. Another explanation might be that the previous studies had a higher frequency of mothers smoking more than 10 cigarettes per day. Since the effect of maternal smoking is most pronounced at this high cigarette consumption [3] and paternal and maternal smoking habits are linked to each other, a high percentage of mothers being heavy smokers might, in a statistical multivariate analysis, weaken the impact of paternal smoking. In our study, we adjusted for maternal smoking based on reliable register data from early pregnancy, when the foetal gonad differentiates [11], [17], [18], [52] and seems most sensitive to harmful effects of toxicants. The details of how the information on paternal smoking was given to the study participants are not known, but we have reasons to believe that underreporting due to shame is less pronounced than the case is for maternal smoking, since the issue of paternal smoking in relation to health of the offspring is less debated than the case is for maternal lifestyle factors.

We expect under- or overestimation of paternal smoking as being non-differential with respect to the outcomes [53] and therefore hardly explaining the statistically significant findings regarding sperm count. Such misclassification would rather lead to an underestimation of a true difference between the groups [53].

The effect of paternal smoking was only statistically significant if the mother was a non-smoker and that of maternal smoking only if the father was a non-smoker. It can not be excluded that the effect of one smoking parent is not significantly different from that of both parents being smokers and these two scenarios are therefore impossible to discriminate in a statistical analysis. In spite of access to MBR data, an underestimation of maternal smoking could underestimate maternal effects, but less likely explain the negative statistical effects of paternal smoking and the absence of maternal effects in paternally exposed men.

For other potential effects of paternal smoking related to the time of pregnancy, some associations with the reproductive function of the offspring have been reported, such as a lower reproductive life span in daughters [54] as well as cryptorchidism [45], [46] and hypospadias [55] in sons, albeit with some inconsistencies [55]–[59]. Our data collection was not optimised in relation to obtaining reliable information about previous and current cryptorchidism, why we cannot exclude this condition as a pathogenetic link between paternal smoking and lower sperm numbers observed by us.

Several studies have suggested that smoking *per se* causes different types of sperm DNA damage [15], [16], [60], and paternal smoking seems independently associated with DNA adducts in embryos [19], childhood cancer in the offspring [31]–[34], [36], pregnancy loss [23], [24], lower pregnancy rate after assisted reproduction [22], non-genital birth defects [25], [26], [28]–[30] as well as with reduced birth weight [37]. Paternal smoking during pregnancy probably highly reflects smoking closely before conception and could thereby exert its effects through mutations or epigenetic changes in the paternal germ line by transmission to the sons. This has been suggested for other paternal exposures and outcomes in the offspring [61]. Such transmission may be due to a lower DNA repair function in male germ cells than in other cells [62]. This is further supported by mutations in spermatogonial stem cells after tobacco smoke exposure [63], by air pollution-induced hypermethylation in sperm DNA [64] as well as air pollution-induced mutations inherited through the male germline [38] in mice [65]. In addition, elevated DNA damage in newborns of fathers who smoked at conception has been reported [21]. However, we found no impact of parental or own smoking on the sperm DFI of our study subjects. This does not exclude that some degree of sperm DNA damage might be detected by use of other techniques than SCSA. Alternative explanations of the association we found between paternal smoking and semen quality in sons, might be that paternal smoking is a marker for other unknown lifestyle-related factors with an effect on sperm number.

Like many previous studies we did find a statistically significant association between maternal smoking and semen quality of the sons, but we only found it in paternally unexposed men. One explanation for this discrepancy might be a low proportion of smoking mothers in Sweden as compared to other countries. Thus, in our cohort the frequency of maternal smoking was 50% lower than the corresponding figure in a Danish study [3]. National discrepancies may not only be linked to the frequency of smoking mothers but may also be related to other life-style factors associated with smoking.

The major strengths of this study are the use of young men from the general population, the register-based data on maternal smoking early in pregnancy, including a comparison with questionnaires to the sons and the more limited use of tobacco in Sweden compared to that in the studies from other countries. The major weak points of this study are the moderate study size, illustrated by groups of 30–40 men with maternal or paternal smoking as the only exposure, and the fact that information on paternal smoking was reported by the sons around 20 years after the exposure period of interest.

## Conclusions

In conclusion, after comparisons between three different exposures and five outcomes, sperm numbers were reduced in young men prenatally exposed to paternal smoking, even after adjustment for own smoking and indoor exposure in childhood, but a reduction was only seen in men of non-smoking mothers. In addition, maternal smoking was associated with a lower sperm concentration in men who were not exposed to prenatal paternal smoking.

## Supporting Information

File S1 **Part A:** Selected question to the examining physician at the genital examination (translated from Swedish). **Part B:** Selected questions from the questionnaire to the participants (translated from Swedish). **Part C.** Extract from paper form filled in by midwife at maternity ward (translated from Swedish).
(DOCX)
