# Supplementary material for: The Impact of Paternal and Maternal Smoking on Semen Quality of Adolescent Men
Source: PLoS One. 2013 Jun 26;8(6):e66766. doi: 10.1371/journal.pone.0066766 (PMC3694111; doi:10.1371/journal.pone.0066766)
Supplement: File S1 — Part A: Selected question to the examining physician at the genital examination (translated from Swedish). Part B: Selected questions from the questionnaire to the participants (translated from Swedish). Part C. Extract from paper form filled in by midwife at maternity ward (translated from Swedish). (DOCX) [file pone.0066766.s001.docx]

**Supporting information**

**A. Extract from the genital examination:**

Varicocele Left: Right:

(0 – none; 1 – 3 grade)

**B. From questionnaire to participants:**

Do you smoke?

No

Yes, cigarettes  number/day_______

Yes, other  what and number/day________

Did your mother smoke when she was pregnant with you?

Don’t know

No

Yes

Did your father smoke when your mother was pregnant with you?

Don’t know

No

Yes

Did any of your parents smoke when you were a child?

Don’t know

No

Yes, indoors

Yes, outdoors

Have you ever from a doctor got to know that you have one or several of following diseases?

Yes: No: Don’t know:

Epididymitis:

Gonorrhoea

Chlamydia

Varicocele

Have you ever had mumps as an adult?

Don’t know  skip next question

No  skip next question

Yes

Were your testicles affected when you had mumps?

Don’t know

No

Yes, one

Yes, both

Have you ever been submitted to a trauma or kick leading to a discoloration och swelling of the scrotum?

Don’t know

No

Yes

Have you ever been surgically treated for varicocele?

Yes  No  Don’t know

Have you ever been treated for cryptorchidism?

Don’t know  No  Yes, operation  Yes, medical treatment

Were you born with any of your testicles outside the scrotum but that spontaneously descended?

Don’t know  No  Yes, right testis  Yes, left testis  Both testes

Have you ever made your partner pregnant?

No

Yes

**C. Question at inscription in maternity ward**

Tobacco at inscription:

No  1-9 cig/day  10 or more cig/day  Snuff
